# Checklist of the parasites of European eel *Anguilla anguilla* (Linnaeus, 1758) (Anguillidae) in Poland

**DOI:** 10.3897/BDJ.8.e52346

**Published:** 2020-06-11

**Authors:** Joanna Dzido, Leszek Rolbiecki, Joanna N. Izdebska, Rafał Bednarek

**Affiliations:** 1 Department of Invertebrate Zoology and Parasitology, Faculty of Biology, University of Gdańsk, Wita Stwosza 59, Gdańsk, Poland Department of Invertebrate Zoology and Parasitology, Faculty of Biology, University of Gdańsk, Wita Stwosza 59 Gdańsk Poland

**Keywords:** biodiversity, eel, fish, parasite, species distribution

## Abstract

The present paper lists all parasite species of the European eel *Anguilla anguilla* (Linnaeus, 1758), recorded in Poland, in both its saltwater and freshwater habitats. The list has been drawn up, based on data acquired since 1844. The majority of included parasite species are presented with fish infection parameters together with data on their developmental stages and occupied microhabitats, localities and dates of collection of the eels themselves. The database includes 62 parasite taxa (including 50 species, nine identified to the genus level and three to higher taxa), representing at least 47 genera and 39 families. The most frequently-noted parasites of the European eel are the cestode *Bothriocephalus claviceps*, the nematodes *Anguillicoloides crassus*, *Camallanus lacustris* and *Raphidascaris acus* and the acanthocephalan *Acanthocephalus lucii*. Four alien species have been noted from this host: *A. crassus*, the monogeneans *Pseudodactylogyrus anguillae* and *Pseudodactylogyrus bini* and the acanthocephalan *Paratenuisentis ambiguus*. The present list includes both new host records and earlier records not included in previous lists of parasites of eels.

## Introduction

The European eel *Anguilla anguilla* (Linnaeus, 1758) is a species of fish with a wide distribution in European waters and one with both very high environmental and economic value. Therefore, there is a pressing need to understand the real and potential treats for eel populations, including such death hazards as parasitoses and their secondary consequences. Since the end of the 20th Century, eel populations have decreased by over 99% due to various factors, such as increased water pollution, climate change, overfishing and dam construction and the species is at risk of extinction. It is currently subject to protection by various forms of conservation, including the Washington Convention (CITES) and is listed as critically endangered by the IUCN Red List of Threatened Species (Dekker 2003, Stone 2003, Freyhof and Kottelat 2010). One significant factor in this decline was the appearance of the nematode *Anguillicoloides crassus*: an alien, invasive parasite which inhabits the swim bladders of eels, resulting in sickness and the disturbance of various vital functions that may prevent the eels from reaching their spawning area and reproducing (Moriarty and Dekker 1997, Lefebvre et al. 2002, Dekker 2003, Kirk 2003, Stone 2003, van Ginneken and Maes 2005). *Anguillicoloides crassus* was probably introduced from Taiwan, where it was associated with its specific host, the Japanese eel; since its introduction, it has spread rapidly throughout the European eel population (Taraschewski et al. 1987, Moravec 1992, Molnár et al. 1993, Münderle et al. 2006).

A comprehensive analysis of the European eel parasite fauna is complicated by its wide geographical distribution and by the poor understanding of the complex biology of the eel. It is therefore often difficult to draw firm conclusions on the infection routes with parasites or participation of the eel in their life cycles. Eel leptocephalus larvae migrate across the Atlantic Ocean to the coasts of Europe; then they metamorphose into glass eel (montée) and move to rivers and lakes, where they mature. Finally, they take part in catadromous migration to the spawning area in the Sargasso Sea (van Ginneken and Maes 2005). Thus, they inhabit different environmental conditions at different stages of ontogenesis; in addition, during the course of their migrations, they may accumulate parasites originating from different areas and further disseminate them. Parasite accumulation is also supported by their longevity and predatory lifestyle, the oldest known specimen in the wild being 85 years old (Dekker et al. 1998). The eel may constitute a significant link in the life cycles of parasites and their distribution in the environment; however, some differences may be attributed to local factors.

Therefore, there is a clear need to better understand the parasite fauna of European eels, its species composition, structural changes and infection level, both on the global and regional scales. Constant parasitological monitoring in all distribution areas would provide a clearer picture of the formation of parasite assemblages across the different parts of the distribution of the eel; it would also allow observation of changes in the parasite ranges and hence, any associated threats. However, being rather local in nature, data on the eel parasite fauna are scattered across a range of publications and reports; in addition, collective analyses are often further complicated by variations in sources or language barrier. Thus far, three checklists of eel parasites have been published concerning various species of *Anguilla*: two of them are from Japan, the second being a revised and updated checklist, while the other concerns the parasites found in *A. anguilla* in Europe and North Africa (Nagasawa et al. 2007, Jakob et al. 2016, Nagasawa and Hirotaka 2017). Out of necessity, the latter list is restricted to available sources from selected countries. Its aim was to provide an overview of the parasite fauna acting as the reference point to future analyses of trends in changes in biodiversity. However, this work did not provide a full picture of the data from Poland, as it included only seven original studies; in contrast, the present checklist includes 59.

Therefore, the objective of this study was to provide a complete, verified list of parasitic Protista and Metazoa found, thus far, in the European eel in Poland.

## Material and methods

The checklist has been drawn up primarily on the basis of published data (55 items) from the area of Poland, including data from the period 1844-2016. It also includes our own unpublished data, marked in the table as "this study", together with examples of data from conference abstracts regarding the occurrence of *A. crassus* or the presence of new records from Poland.

For the majority of species, additional data have been provided if included in the source publications: infection parameters such as prevalence (P), mean intensity, intensity range and abundance, as well as the developmental stages of the parasites and their microhabitats. The infection parameters were calculated, based on data included in original studies by means of unification, where possible. Information on dates of fish collection, as well as the geographical location (with GPS coordinates in the Suppl. material 1), have been also included.

The species were arranged in taxonomic and then alphabetical order. The Protista taxonomy follows Lom and Dyková (1992); for Trematoda, Gibson et al. (2002) and Jones et al. (2005); for Nematoda, Moravec (2013) and Nadler et al. (2005); for Cestoda, Kahlil et al. (1994); for Acanthocephala, Amin (2013); the taxonomy for Myxozoa, Monogenea, Arthropoda, Annelida and Mollusca follows the WORMS database (WoRMS Editorial Board 2020). As some taxa have been subject to revision over the years, valid and verified species names were used in the list. For instance, Szidat (1944) considered *Sphaerostomum bramae* (Müller, 1776), a trematode from the study of Markowski (1933) as *Plagioporus angulatus*. Furthermore, *Spironucleus mobilis* Wierzbicka & Einszporn-Orecka, 1986 is currently a synonym of *Spironucleus anguillae* (Lom and Dyková 1992), *Trichophrya piscium* Buetschli, 1889 is *Capriniana piscium* (Svobodová et al. 2009), *Sphaerospora sphaerocapsularae* Wierzbicka 1986 is now *Ortholinea sphaerocapsularae* (Sitjà-Bobadilla and Alvarez-Pellitero 1994), *Sphaerospora anguillae* Wierzbicka, 1986 is a synonym of *S. gilsoni* (Wierzbicka 1994), *Ascaris labiata* Rudolphi, 1809 is *Raphidascaris acus*, *Anguillicola crassus* Kuwahara, Niimi & Itagaki, 1974 is currently known as *Anguillicoloides crassus* (Moravec 2006) and *Contracaecum aduncum* (Rudolphi, 1802) is *Hysterothylacium aduncum*. Bielecki et al. (2011) believe that *Cystobranchus respirans* (Troschel, 1850) is *Piscicola respirans*. In addition, it is known that "*Diplostomum spathaceum*" includes more than one species; there are several species of *Diplostomum* difficult to identify without using molecular methods (Niewiadomska 2003, Georgieva et al. 2013). Likewise, there is a possibility of wrong species identifitaciton in the case of *Pomphorhynchus laevis*, due to morphological similarity to *P. tereticollis* which occurs sympatrically (Špakulová et al. 2011, Hohenadler et al. 2018).

## Results and Discussion

A total of 62 taxa have been recorded from the European eel in Poland, of which 11 are Protista (eight species and three identified at the genus level), including five Ciliophora (one identified at the genus level as *Apiosoma*) and two Apicomplexa. Of the 51 representatives of Metazoa (42 identified as species, six to genus level, three to higher taxa), six were Myxozoa, nine Trematoda, five Monogenea (one identified at the genus level – *Dactylogyrus* and one as Monogenea), five Cestoda (one identified as Cestoda gen. sp. Pseudophyllidearum larvae), ten Nematoda, eight Acanthocephala, two Annelida, three Arthropoda, one Mollusca (identified as Unionidae). In addition to the species mentioned in Table 1, further data on the occurrence of *Acanthocephalus clavula* and *Corynosoma semerme* in the European eel were given in overview studies on the parasites of the Polish ichthyofauna (Pojmańska et al. 2007, Popiołek 2016); however, this information was not included in the analysed original studies.

For comparison, the list of European eel parasites (data until 2009) from 30 countries in Europe and North Africa specifies 161 parasitic taxa (129 identified to species), of which 146 were metazoans and 15 were Protista (Jakob et al. 2016): *Epieimeria anguillae* and *Eimeria anguillae, which were* listed as two distinct species, are now considered to be the same taxon in the genus *Eimeria*, according to Benajiba et al. (1994). Similarly to the Polish study, digenetic trematodes (39 species) and nematodes (38 species) formed the most species-rich groups.

Twenty six parasite species, included in the present list, are also present in Jakob et al. (2016). However, that list does not include many parasite species and localities from Poland and does not reflect the actual distribution of the parasites. For example, Poland was not given as the area of occurrence for 23 parasite species (*Trypanosoma granulosum*, *Apiosoma* sp., *Ichthyophthirius multifiliis*, *Trichodina jadranica*, *Trichodinella epizootica*, *Capriniana piscium*, *Rhabdospora thelohani*, *Henneguya psorospermica*, *Myxobolus portucalensis*, *Myxidium giardi*, *Zschokkella stettinensis*, *Ortholinea sphaerocapsularae*, *Bunodera luciopercae*, *Dactylogyrus* sp., *Triaenophorus nodulosus*, *Cystidicola farionis*, *Spinitectus inermis*, *Hysterothylacium aduncum*, *Paraquimperia tenerrima*, *Corynosoma strumosum*, *Echinorhynchus gadi*, *Echinorhynchus truttae* and *Paratenuisentis ambiguus*) in Jakob et al. (2016). In addition, a number of groups and species, included in the present list, were absent from Jakob et al. (2016). For instance, the group of protists from Poland has now been expanded to include *Capriniana piscium* and *Ortholinea sphaerocapsularae* and a representative of *Apiosoma* without species identification. *Rhabdospora thelohani* is also mentioned; however, considerable controversy exists as to whether this species is indeed a representative of apicomplexan parasites or a host “rodlet cell” (Davies and Ball 1993). In addition, amongst the Metazoa, the new list has been enriched with the addition of *Henneguya psorospermica*, *Cystidicola farionis* and an unidentified *Dactylogyrus* species for Poland, as well as unidentified cestodes found by Sołtyńska (1964). The present list includes a number of new localities for eel parasites, previously unpublished (marked in Table 1 as "this study")

In comparison, only nine species of parasites were recorded for European eels in Japan, as well as seven taxa identified at genus level and an unidentified Monogenea. However, these eels also included parasites, thus far unknown from the European *A. anguilla* (*Cryptobia* spp., *Ichthyobodo* spp., *Gyrodactylus anguillae*, *Lernaea cyprinacea*) (Nagasawa et al. 2007, Nagasawa and Hirotaka 2017). This confirms the possibility that regional differences may exist, not only with regard to the level of infection, but also in the composition and species diversity of the parasite fauna.

Within the parasitofauna of eel, the greatest repeatability between different distribution areas is exhibited by the parasites specific to the genus *Anguilla* (e.g. the nematode *A. crassus*, the cestodes *Bothriocephalus claviceps* and *Proteocephalus macrocephalus* or the trematode *Deropristis inflata*), but also certain widely-distributed species with large ranges of hosts, such as the trematode *Diplostomum spathaceum* s. l., the leech *Piscicola geometra* or the copepod *Ergasilus sieboldi*. It is in this area that the number of records of alien and invasive parasites increases for the European eel; for example, *A. crassus*, which was introduced to Europe in 1982 and recorded in Poland in 1988 (Koops and Hartmann 1989, Własow 1991, Bystydzieńska et al. 2005) or *Pseudodactylogyrus anguilla* and *P. bini*, recorded in Poland in 1995 by Dzika et al. (1995). It is also important to note the presence of a new, potentially invasive species, the acanthocephalan *Paratenuisentis ambiguus*, originally a parasite of *Anguilla rostrata* (Lesueur, 1817), which was first recorded in a European eel from Europe in 1980 and later in Poland by Morozińska-Gogol (2008).

The dispersal of parasites, their increased prevalence and level of infection are linked not only to the life history of eels and their migrations. Parasite infection in local eel populations can also transmitted through stocking material. For instance, protozoan *Trichodina fultoni* (100% infection) was found in rearing glass eel imported to Poland from France in 1971 (Markiewicz and Migała 1980). In addition, the monogenean *P. anguillae* and the nematode *A. crassus* were found in eels originating from the stocking material for the Vistula Lagoon in 2006 (Rolbiecki et al. 2008). What is more, of the eels originating from farming facilities located in the Warmian-Masurian Voivodeship (northeast Poland) and studied in the period 2010-2014, 77.2% were found to have *Pseudodactylogyrus* spp.; *Trichodina* spp. and *I. multifiliis* were typical parasites at the early rearing stage (Terech-Majewska et al. 2016).

With their resources already being considerably depleted and the growing number threats to eel populations, there has been a growing interest in their parasitic fauna; this growth has been accompanied by a greater need to carry out regular monitoring of parasitological threats, especially actual or potentially pathogenic species, including alien and invasive species. However, such data have to be constantly supplemented and verified with new records of parasites in different parts of distribution of this host. As such research would be complicated by the degree of data scatter, the best solution would be to create a web-based database, supplemented and coordinated by scientific centres from different countries.

## Supplementary Material

1C83DB9F-B404-538B-B560-569DAF69D4CC 10.3897/BDJ.8.e52346.suppl1 Supplementary material 1 GPS coordinates of collection sites Data type Table File: oo_416475.docx https://binary.pensoft.net/file/416475 Joanna Dzido, Leszek Rolbiecki, Joanna N. Izdebska, Rafał Bednarek

## Figures and Tables

**Table 1.** Checklist of the protozoan and metazoan parasites of *Anguilla anguilla* from Poland. The parameters have been provided with accuracy of original data; if the parameter was not given in the original work, it was marked with a dash (-). Parasite life stage: A – adults, e – eggs, F – female, L – larvae, M – male, met – metacercariae, pA – preadults, pl – plasmodia. Microhabitat: sb – swim bladder, ub – urinary bladder. Intensity: (+) – single to not numerous, (++) – rather numerous to numerous, (+++) – very numerous, (++++) – mass occurrence.

Parasite life stage	Micro habitat	P [%]	Intensity range (mean)	Abun dance	Locality	Material collection year	References
METAMONADA							
**Family Hexamitidae**							
*Spironucleus anguillae* Einszporn-Orecka, 1979							
-	^1^	-	^2^	-	Szczecin Lagoon	1974	Einszporn-Orecka 1979
-	intestine	1.9 ^3^	(+)-(++)	-	Szczecin Lagoon	1982-83	Wierzbicka and Einszporn-Orecka 1986, Wierzbicka and Orecka-Grabda 1994
-	intestine	4.2	(++)	-	Oder mouth	1982-83	Wierzbicka and Einszporn-Orecka 1986, Wierzbicka and Orecka-Grabda 1994
EUGLENOZOA							
**Family Trypanosomatidae**							
*Trypanosoma granulosum* Laveran & Mesnil, 1909							
trypomastigote	blood	24	(0.2-16.0) ^4^	-	Lake Siecino	1970-73	Orecka-Grabda and Wierzbicka 1996
trypomastigote	blood	68	(2.2-16.2) ^4^	-	Lake Dąbie	1970-73	Orecka-Grabda and Wierzbicka 1996
-	blood	-	-	-	Szczecin Lagoon, Lake Dąbie	1970-73	Orecka-Grabda 1986
-	^5^	46.9 ^3^	(+)-(++)	-	Szczecin Lagoon	1982-83	Wierzbicka and Orecka-Grabda 1994
-	^5^	95.8	(+)-(++)	-	Oder mouth	1982-83	Wierzbicka and Orecka-Grabda 1994
-	-	50-60	-	-	Lake Śniardwy	1989	Własow et al. 1991
-	-	50	-	-	Lake Mamry	1990	Własow et al. 1991
-	blood	100	variable	-	River Rega	2001-02	Rząd et al. 2007
*Trypanosoma* sp.							
-	blood	24	-	-	Lake Ińsko	1993	Rząd and Pilecka-Rapacz 2002
CILIOPHORA							
**Family Epistylididae**							
*Apiosoma* sp.							
-	-	^6^	-	-	Lake Dąbrowa Wielka	1990-91	Własow et al. 1991
**Family Ichthyophthiriidae**							
*Ichthyophthirius multifiliis* (Fouquet, 1876)							
-	gills, skin	-	-	-	River Darłówka	-	Grabda 1971
-	gills	3.1 ^3^	(+)	-	Szczecin Lagoon	1982-83	Wierzbicka and Orecka-Grabda 1994
**Family Trichodinidae**							
*Trichodina jadranica* Raabe, 1958							
-	gills	68.6 ^3^	(+)-(++++)	-	Szczecin Lagoon	1982-83	Wierzbicka and Orecka-Grabda 1994
-	gills	66.7	(+)	-	Oder mouth	1982-83	Wierzbicka and Orecka-Grabda 1994
*Trichodina* sp.							
-	-	30	-	-	Lake Niegocin	1989	Własow et al. 1991
-	-	8	-	-	Lake Mamry	1990	Własow et al. 1991
-	-	^6^	-	-	Lake Dąbrowa Wielka	1990-91	Własow et al. 1991
*Trichodinella epizootica* (Raabe, 1950)							
-	gills	62.9 ^3^	(+)-(+++)	-	Szczecin Lagoon	1982-83	Wierzbicka and Orecka-Grabda 1994
-	gills	41.7	(+)-(+++)	-	Oder mouth	1982-83	Wierzbicka and Orecka-Grabda 1994
-	-	3.3	1-4 (2.5)	-	Vistula Lagoon	2005	Rolbiecki and Rokicki 2006
**Family Trichophryidae**							
*Capriniana piscium* (Bütschli, 1889)							
-	gills	4.2	(+++)	-	Oder mouth	1982-83	Wierzbicka and Orecka-Grabda 1994
APICOMPLEXA							
**Family Eimeriidae**							
*Eimeria anguillae* Léger & Hollande, 1922							
oocyst	intestine	11.9 ^3^	(+)-(++++)	-	Szczecin Lagoon	1982-83	Wierzbicka and Orecka-Grabda 1994
oocyst	intestine	20.8	(+)-(+++)	-	Oder mouth	1982-83	Wierzbicka and Orecka-Grabda 1994
Family uncertain							
*Rhabdospora thelohani* Laguessé, 1895							
-	-	20	-	-	Lake Niegocin	1989	Własow et al. 1991
-	-	50-60	-	-	Lake Śniardwy	1989	Własow et al. 1991
-	-	17	-	-	Lake Mamry	1990	Własow et al. 1991
-	-	49	-	-	Lake Dąbrowa Wielka	1990-91	Własow et al. 1991
MYXOZOA							
**Family Myxidiidae**							
*Myxidium giardi* Cépède, 1906							
spores	^7^	79.9 ^3^	(+)-(+++)	-	Szczecin Lagoon	1982-83	Wierzbicka and Orecka-Grabda 1994
spores	^7^	100	(+)-(++++)	-	Oder mouth	1982-83	Wierzbicka and Orecka-Grabda 1994
-	gills	20	(+)-(++)	-	Lake Miedwie	1997-99	Sobecka and Piasecki 2002
-	-	21.1	(++)-(+++)	-	Vistula Lagoon	2005	Rolbiecki and Rokicki 2006
*Zschokkella stettinensis* Wierzbicka, 1987							
spores	ub	48.65	(+)-(+++)	-	Szczecin Lagoon, Lake Dąbie	1983, 1985	Wierzbicka 1987
-	ub	11.3 ^3^	(+)-(+++)	-	Szczecin Lagoon	1982-83	Wierzbicka and Orecka-Grabda 1994
-	ub	50.0	(+)-(+++)	-	Oder mouth	1982-83	Wierzbicka and Orecka-Grabda 1994
**Family Myxobolidae**							
*Henneguya psorospermica* Thélohan, 1895							
-	-	^6^	-	-	Lake Dąbrowa Wielka	1990-91	Własow et al. 1991
*Myxobolus portucalensis* Saraiva & Molnar, 1990							
pl, spores	^8^	16.9	(+)-(++)	-	Szczecin Lagoon	1982-83	Wierzbicka and Orecka-Grabda 1996
pl, spores	^8^	29.2	(+)-(++)	-	Skolwiński Canal	1982-83	Wierzbicka and Orecka-Grabda 1996
pl, spores	^8^	38.5	(+)-(++)	-	Lake Dąbie	1985	Wierzbicka and Orecka-Grabda 1996
**Family Ortholineidae**							
*Ortholinea sphaerocapsularae* (Wierzbicka, 1986)							
spores	ub	7.69	(++)	-	Lake Dąbie	1985	Wierzbicka 1986b
**Family Sphaerosporidae**							
*Sphaerospora gilsoni* (Debaisieux, 1925)							
pl, spores	ub	13.2 ^3^	(+)-(+++)	-	Szczecin Lagoon	1982-83	Wierzbicka and Orecka-Grabda 1994
pl, spores	ub	87.5	(+)-(++++)	-	Oder mouth	1982-83	Wierzbicka and Orecka-Grabda 1994
spores	ub	87.5	(+)-(+++)	-	Szczecin Lagoon	1983	Wierzbicka 1986a
PLATYHELMINTHES: TREMATODA							
**Family Allocreadiidae**							
*Bunodera luciopercae* (Müller, 1776)							
A	-	1.1	2	0.023	Vistula Lagoon	2005	Rolbiecki and Rokicki 2006
**Family Azygiidae**							
*Azygia lucii* (Müller, 1776)							
-	stomach	0.63 ^3^	1 (1)	0.006 ^3^	Szczecin Lagoon	1982-83	Orecka-Grabda and Wierzbicka 1994
**Family Deropristidae**							
*Deropristis inflata* (Molin, 1859)							
A	intestine	18.5 ^3^	1-12 (3.8 ^3^)	0.70 ^3^	Baltic Sea (n. Chłapowo), Puck Bay	1930-31	Markowski 1933
-	intestine	17.6 ^3^	2-30 (13)	-	Gulf of Gdańsk	1967-71	Rokicki 1975
-	-	3	1 (1)	0.03 ^3^	Lake Dąbie	1971	Seyda 1973
-	intestine	3.8 ^3^	1-63 (14.83)	0.56 ^3^	Szczecin Lagoon	1982-83	Orecka-Grabda and Wierzbicka 1994
A	intestine	1.43	2-4	0.042	Dead Vistula	1982-90	Sulgostowska 1993
A	intestine	12.17	1-600	2.190	Gulf of Gdańsk	1982-90	Sulgostowska 1993
A	intestine	42.15	1-200	16.016	Baltic Sea (n. Władysławowo)	1982-90	Sulgostowska 1993
-	intestine	1.14	2 (2.00)	0.02	Lake Łebsko	2000-06	Morozińska-Gogol 2011
-	-	0.4	1 (1)	0.002 ^3^	Vistula Lagoon	2001-02	Bystydzieńska et al. 2005
-	-	0.7	1 (1)	0.007 ^3^	Puck Bay	2002	Bystydzieńska et al. 2005
A	-	2.2	2-6 (3)	0.09 ^3^	Vistula Lagoon	2005	Rolbiecki and Rokicki 2006
**Family Diplostomidae**							
*Diplostomum spathaceum* s. l. (Rudolphi, 1819)							
met	-	10	1 (1)	0.10 ^3^	Oder (n. Stołczyn)	1971	Seyda 1973
met	-	31	1-4 (1.6 ^3^)	0.50 ^3^	Szczecin Lagoon	1971	Seyda 1973
met	-	6	1 (1)	0.10 ^3^	Lake Dąbie	1971	Seyda 1973
*Diplostomum* spp.							
met	-	9.1	2 ^3^ (2.0 ^3^)	0.2	Lake Dgał Wielki	1979-84	Grabda-Kazubska et al. 1987
met	-	^6^	-	-	Lake Mamry	1990	Własow et al. 1991
met	-	19	-	-	Lake Dąbrowa Wielka	1990-91	Własow et al. 1991
met	eyes	3.41	1-3 (3.67)	0.13	Lake Łebsko	2000-06	Morozińska-Gogol 2007, Morozińska-Gogol 2011
met	eyes	51.1	1-88 (6.8)	-	Puck Bay	2002	Bystydzieńska et al. 2005
met	-	8.9	1-2 (1.4)	-	Vistula Lagoon	2005	Rolbiecki and Rokicki 2006
met	eye lens	14.3 ^9^	6 (6.0)	0.85	Rzeka Łeba	2014-15	This study
*Tylodelphys clavata* (Nordmann, 1832)							
met	vitreous humour	3	1 (1)	0.03 ^3^	Szczecin Lagoon	1971	Seyda 1973
met	vitreous humour	3	1 (1)	0.03 ^3^	Lake Dąbie	1971	Seyda 1973
**Family Hemiuridae**							
*Brachyphallus crenatus* (Rudolphi, 1802)							
-	-	5.9 ^3^	9	0.53 ^3^	Gulf of Gdańsk	1967-71	Rokicki 1975
-	-	0.2	1 (1)	0.002 ^3^	Vistula Lagoon	2001-02	Bystydzieńska et al. 2005
**Family Opecoelidae**							
*Plagioporus angulatus* (Dujardin, 1845)							
A	intestine	7.4 ^3^	1-3 (2)	0.15 ^3^	Baltic Sea (n. Chłapowo)	1930-31	Markowski 1933
**Family Strigeidae**							
*Ichthyocotylurus platycephalus* (Creplin, 1825)							
met	stomach ^10^	0.63 ^3^	3 (3)	0.02 ^3^	Szczecin Lagoon	1982-83	Orecka-Grabda and Wierzbicka 1994
met	-	2.2	3 (3)	0.07 ^3^	Vistula Lagoon	2005	Rolbiecki and Rokicki 2006
PLATYHELMINTHES: MONOGENEA							
**Family Dactylogyridea**							
*Dactylogyrus* sp.							
-	gills	30	(1)	-	Lake Miedwie	1997-99	Sobecka and Piasecki 2002
**Family Pseudodactylogyridae**							
*Pseudodactylogyrus anguillae* (Yin & Sproston, 1948)							
-	gills	-	-	3.46 ^3^	Lake Strażyn	-	Dzika et al. 1995
-	gills	90	(11.6)	10.4	Lake Dębno	1994-95	Dzika 1999
-	gills	^11^	(1.82-9.25)	0.14-4.78	Rivers Radew, Rega, Wieprza	1999-2003	Sobecka and Pilecka-Rapacz 2003
-	gills	1.13	3 (3.0)	0.03	Lake Łebsko	2000-06	Morozińska-Gogol 2009, Morozińska-Gogol 2011
-	gills	100 ^9^	6-16 (11)	11.0 ^3^	Puck Bay	2002	Bystydzieńska et al. 2005
*Pseudodactylogyrus bini* Kikuchi, 1929							
-	gills	-	-	2.07 ^3^	Lake Strażyn	-	Dzika et al. 1995
-	gills	71	(18.1)	16.2	Lake Dębno	1994-95	Dzika 1999
-	gills	^11^	(0.73-3.5)	0.12-1.95	River Radew, Rega, Wieprza	1999-2003	Sobecka and Pilecka-Rapacz 2003
*Pseudodactylogyrus* sp.							
-	gills	1.9 ^3^	1 (1)	0.02 ^3^	Szczecin Lagoon	1982-83	Orecka-Grabda and Wierzbicka 1994
Monogenea n. det.							
-	-	17	-	-	Lake Dąbrowa Wielka	1990-91	Własow et al. 1991
PLATYHELMINTHES: CESTODA							
**Family Bothriocephalidae**							
*Bothriocephalus claviceps* (Goeze, 1782)							
A	intestine	22.2 ^3^	2-3 (2.5 ^3^)	0.55 ^3^	Baltic Sea (n. Chłapowo), Puck Bay	1930-31	Markowski 1933
A	-	-	-	-	Lake Gołdapiwo, Lake Mamry	1954-58	Jarecka 1959
A	intestine	8.3 ^3^	1 (1)	0.08 ^3^	Puck Bay	1959	Sołtyńska 1964
-	-	25	1-11 (4.2 ^3^)	1.05 ^3^	Oder (n. Stołczyn)	1971	Seyda 1973
-	-	37	1-14 (4.9 ^3^)	1.84 ^3^	Szczecin Lagoon	1971	Seyda 1973
-	-	23	2-13 (3.7 ^3^)	0.84 ^3^	Lake Dąbie	1971	Seyda 1973
-	-	9.1	3 ^3^ (3 ^3^)	0.27	Lake Dgał Wielki	1979-84	Grabda-Kazubska et al. 1987
-	intestine	35.2 ^3^	1-5 (1.1 ^3^)	0.40 ^3^	Szczecin Lagoon	1982-83	Orecka-Grabda and Wierzbicka 1994
-	intestine	29.2	1-3 (1.4 ^3^)	0.42 ^3^	Skolwiński Canal	1982-83	Orecka-Grabda and Wierzbicka 1994
immature, A	intestine	10.00	1-35	0.807	Dead Vistula	1982-90	Sulgostowska 1993
immature, A	intestine	8.36	1-10	0.183	Gulf of Gdańsk	1982-90	Sulgostowska 1993
immature, A	intestine	11.57	1-18	0.371	Baltic Sea (n. Władysławowo)	1982-90	Sulgostowska 1993
-	-	^6^	-	-	Lake Dąbrowa Wielka	1990-91	Własow et al. 1991
-	intestine	12.5	1-6 (2.27)	0.28	Lake Łebsko	2000-06	Morozińska-Gogol 2011
-	-	0.8	1-2 (1.5)	-	Vistula Lagoon	2001-02	Bystydzieńska et al. 2005
-	-	30.1	1-11 (2.8)	-	Puck Bay	2002	Bystydzieńska et al. 2005
A	intestine	15.2	1-14 (3.4)	0.52	Lake Wdzydze	2004	This study
A	-	18.9	1-3 (1.3)	-	Vistula Lagoon	2005	Rolbiecki and Rokicki 2006
**Family Proteocephalidae**							
*Proteocephalus macrocephalus* (Creplin, 1825)							
A	intestine	22.2 ^3^	1-20 (5.0 ^3^)	1.10 ^3^	Baltic Sea (n. Chłapowo), Puck Bay	1930-31	Markowski 1933
plerocercoid	intestine	8.3 ^3^	1 (1)	0.08 ^3^	Puck Bay	1959	Sołtyńska 1964
-	intestine	4.1 ^3^	1-19	-	Gulf of Gdańsk	1967-71	Rokicki 1975
-	-	10	1-3 (2.0 ^3^)	0.20 ^3^	Oder (n. Stołczyn)	1971	Seyda 1973
-	-	22	1-10 (2.9 ^3^)	0.62 ^3^	Szczecin Lagoon	1971	Seyda 1973
-	-	26	1-4 (4.5 ^3^)	1.16 ^3^	Lake Dąbie	1971	Seyda 1973
-	intestine	39.0 ^3^	1-33 (3.3 ^3^)	1.30 ^3^	Szczecin Lagoon	1982-83	Orecka-Grabda and Wierzbicka 1994
-	intestine	16.7	1-2 (1.5 ^3^)	1.50 ^3^	Skolwiński Canal	1982-83	Orecka-Grabda and Wierzbicka 1994
immature, A	intestine	15.71	1-5	0.300	Dead Vistula	1982-90	Sulgostowska 1993
immature, A	intestine	10.12	1-25	0.253	Gulf of Gdańsk	1982-90	Sulgostowska 1993
immature, A	intestine	24.79	1-12	0.743	Baltic Sea (n. Władysławowo)	1982-90	Sulgostowska 1993
-	intestine	27.27	1-15 (3.75)	1.02	Lake Łebsko	2000-06	Morozińska-Gogol 2011
-	-	8.4	1-22 (3.8)	-	Vistula Lagoon	2001-02	Bystydzieńska et al. 2005
-	-	23.3	1-42 (4.8)	-	Puck Bay	2002	Bystydzieńska et al. 2005
A	-	31.1	1-5 (2.5)	-	Vistula Lagoon	2005	Rolbiecki and Rokicki 2006
A	intestine	20.0 ^9^	2 (2.0)	0.40	River Szkarpawa	2014-16	This study
*Proteocephalus* sp.							
juvenile	-	16.7	1-6 (2.2)	-	Vistula Lagoon	2005	Rolbiecki and Rokicki 2006
**Family Triaenophoridae**							
*Triaenophorus nodulosus* (Pallas, 1781)							
-	intestine	20.0 ^3,9^	1	0.203	Vistula (n. Warszawa)	1924-25	Dąbrowska 1970
Family n. det.							
Cestoda gen. sp. Pseudophyllidarum larvae							
L	intestine	8.3 ^3^	1 (1)	0.08 ^3^	Puck Bay	1959	Sołtyńska 1964
NEMATODA							
**Family Anguillicolidae**							
*Anguillicoloides crassus* (Kuwahara, Niimi & Itagaki, 1974)							
-	sb	75	-	-	Vistula Lagoon	1988	Grawiński 1994
-	sb	80	-	-	Lakes Przywłoczne, Skąpe, Wielewickie	1989	Grawiński 1994
-	-	68.3	1-25	-	Vistula Lagoon	1988-90	Rolbiecki et al. 1996
-	-	2.7 ^3^	1-2	-	Lake Niegocin	1989	Własow et al. 1991
-	-	70.0 ^3^	5-25	-	Goczałkowicki Reservoir	1989-90	Własow et al. 1991
-	-	2.7 ^3^	2-33	-	Lake Mamry	1990	Własow et al. 1991
-	-	-	-	-	^12^	-	Własow 1991
L2-L4, pA, A	sb	78.3	1-204600	-	Lake Strażyn	1993	Własow et al. 1991
L2-L4, pA, A	sb	25	1-102	-	reservoir near the village of Gaj	1993	Własow et al. 1991
-	-	100	15-20	-	Vistula Lagoon	1993	Grawiński 1994
L, A	sb	78.7	1-15	-	Lake Ińsko	1993	Orecka et al. 1995
-	sb	88.7	1-15	-	Lake Ińsko	1993	Rząd and Pilecka-Rapacz 2002
juvenile, F	sb	23.4 ^3^	0-36	-	Szczecin Lagoon	1993-94	Garbacik-Wesołowska et al. 1994
juvenile, F	sb	69.0	0-22	-	Lake Łętowskie	1994	Garbacik-Wesołowska et al. 1994
juvenile, F	sb	23.1 ^3^	0-10	-	Pomeranian Bay	1994	Garbacik-Wesołowska et al. 1994
L	sb	70	1-35	-	Szczecin Lagoon	1994-96	Rząd and Pilecka-Rapacz 2001
-	sb	37.5 ^9^	3-8 (5.0)	-	Dead Vistula	1996	Rolbiecki and Rokicki 2005
-	sb	37.5	5-16 (7.3)	-	Lake Druzno	1997	Rolbiecki and Rokicki 2005
e, L4, pA, A	sb, intestine	41.9	(3.0)	-	Gulf of Gdańsk	1997-98	Rolbiecki et al. 2000
-	sb	100	3-44 (8.2)	6.7	Lake Miedwie	1997-99	Sobecka and Piasecki 2002
L3, L4, A	sb	33.3	1-7	-	River Wieprza (near Darłowo)	1999	Pilecka-Rapacz 2001
L3, L4, A	sb	40	1-10	-	River Rega (near Trzebiatów)	1999	Pilecka-Rapacz 2001
-	sb	59.1	1-11 (1.7)	-	River Rega (Lake Rejowice)	1999-2003	Pilecka-Rapacz and Sobecka 2004
-	sb	41.7	1-8 (1.3)	-	River Wieprza (nearDarłowo)	1999, 2001	Pilecka-Rapacz and Sobecka 2004
-	sb	65.6	1-12 (2.1)	-	River Radew	2000-01	Pilecka-Rapacz and Sobecka 2004
A, L	sb	68.18	1-27 (5.82)	3.97	Lake Łebsko	2000-06	Morozińska-Gogol 2005, Morozińska-Gogol 2007, Morozińska-Gogol 2009, Morozińska-Gogol 2011
-	sb	66.7	11-24 (17.5)	-	Lake Bukowo	2000-07	Morozińska-Gogol 2009
-	sb	100	5-11 (8.0)	-	Lake Kopań	2000-07	Morozińska-Gogol 2009
	-	75	1-58 (10)	-	Vistula Lagoon	2001-02	Bystydzieńska et al. 2005
L4, L5, A	sb	75.9 ^3^	1-11 (3.2)	-	River Rega	2001-02	Rząd et al. 2007
-	-	74.4	1-62 (8.3)	-	Puck Bay	2002	Bystydzieńska et al. 2005
L2-L4, A, e	sb	79.3	1-46 (7.2)	5.7	Lake Wdzydze	2002-05	Rolbiecki 2008
-	sb	100 ^9^	6-18 (12)	-	Lake Raduńskie Dolne, Lake Raduńskie Górne	2004	Rolbiecki and Rokicki 2005
-	sb	58.3	2-12 (6.0)	-	Dead Vistula	2004	Rolbiecki and Rokicki 2005
L3, L4, A, e	sb	67.8	1-37 (4.2)		Vistula Lagoon	2005	Rolbiecki and Rokicki 2006
L2, L4, A, e	sb	65.2	1-20 (5.5)	3.6 ^3^	Lake Ostrzyckie	2005-07	Rolbiecki 2011
-	-	-	-	-	Lake Kuc	2006-07	Jeżewski et al. 2007
L2, A, e	sb	50.0	1-12 (3.8)	1.9 ^3^	Lake Żarnowieckie	2006-08	Rolbiecki 2011
L2, A, e	sb	40.0	2-3 (3.0)	1.2 ^3^	Lake Raduńskie Dolne, Lake Raduńskie Górne	2006-08	Rolbiecki 2011
L4, A	sb	28.6 ^9^	4-6 (5.0)	1.4 ^3^	Vistula (near Tczew)	2007	Rolbiecki 2011
L3, A	sb, intestine wall	100 ^9^	3-6	4.5 ^3^	Lake Żarnowieckie	2007	Rolbiecki 2011
pA, A	sb	92.9	1-49 (10.8)	-	Szczecin Lagoon	-	Popielarczyk et al. 2012
pA, A	sb	64.7	1-5 (1.9)	-	Lake Dąbie	-	Popielarczyk et al. 2012
pA, A	sb	64.2	3-62 (18.5)	-	Lake Bukowo	-	Popielarczyk et al. 2012
pA, A	sb	100 ^9^	1-50 (13.6)	-	Lake Łebsko	-	Popielarczyk et al. 2012
pA, A	sb	80.0 ^9^	3-16 (8.0)	-	Lake Gardno	-	Popielarczyk et al. 2012
pA, A	sb	85.7	1-23 (7.2)	-	Lake Resko	-	Popielarczyk et al. 2012
pA, A	sb	76.9	1-55 (11.6)	-	Lake Jamno	-	Popielarczyk et al. 2012
pA	sb	50.0	1-7 (3.2)	-	Oder	-	Popielarczyk et al. 2012
pA, A	sb	71.4	1-6 (3.4)	-	Vistula	-	Popielarczyk et al. 2012
pA	sb	62.5	1-4 (1.7)	-	River Węgorapa	-	Popielarczyk et al. 2012
pA, A	sb	66.7	1-7 (2.6)	-	River Drwęca	-	Popielarczyk et al. 2012
A	sb	100 ^9^	7 (7.0)	7.0	River Piaśnica	2014-16	This study
A, L4	sb	40.0 ^9^	6-9 (7.5)	3.0	River Szkarpawa	2014-16	This study
A	sb	42.9 ^9^	2-4 (3.0)	1.28	Lake Sarbsko	2016	This study
**Family Camallanidae**							
*Camallanus lacustris* (Zoega, 1776)							
-	intestine	20.0 ^3,9^	1 (1.0)	0.20 ^3^	Vistula (near Warszawa)	1924-25	Dąbrowska 1970
-	intestine	100 ^3,9^	87 (87.0 ^3^)	87.0 ^3^	Lake Wdzydze	1958	Grabda et al. 1961
-	intestine	-	-	-	Lake Gardno, River Nogat (near Tczew), Lake Kalwa	-	Grabda 1971
-	-	15	1-4 (2.7 ^3^)	0.40 ^3^	Oder (near Stołczyn)	1971	Seyda 1973
-	-	3	12 (12)	0.39 ^3^	Lake Dąbie	1971	Seyda 1973
-	-	45.5	(4.2 ^3^)	1.9	Lake Dgał Wielki	1979-84	Grabda-Kazubska et al. 1987
A	intestine	3.1 ^3^	1-7 (3.4 ^3^)	0.11 ^3^	Szczecin Lagoon	1982-83	Orecka-Grabda and Wierzbicka 1994
L4, A	intestine	5.00	1-3	0.057	Dead Vistula	1982-90	Sulgostowska 1993
L4, A	intestine	2.93	1-20	0.152	Gulf of Gdańsk	1982-90	Sulgostowska 1993
-	-	^7^	-	-	Lake Dabrowa Wielka	1990-91	Własow et al. 1991
-	intestine	-	-	-	Lake Ińsko	1993	Rząd and Pilecka-Rapacz 2002
-	intestine	10	1 (1)	-	Lake Miedwie	1997-99	Sobecka and Piasecki 2002
-	intestine	3.41	1-2 (1.67)	0.06	Lake Łebsko	2000-06	Morozińska-Gogol 2011
-	-	3.8	1-7 (2.0)	-	Puck Bay	2002	Bystydzieńska et al. 2005
A	stomach	2.2	2	0.04	Lake Wdzydze	2004	This study
A	-	2.2	2 (2)	0.04 ^3^	Vistula Lagoon	2005	Rolbiecki and Rokicki 2006
A	intestine	25.0 ^9^	7 (7.0)	1.75	Lake Kłodno	2015	This study
*Camallanus truncatus* (Rudolphi, 1814)							
-	-	3	1 (1)	0.033	Szczecin Lagoon	1971	Seyda 1973
L4	-	0.29	1-3	0.005	Gulf of Gdańsk	1982-90	Sulgostowska 1993
A	-	2.2	2-4 (3)	0.073	Vistula Lagoon	2005	Rolbiecki and Rokicki 2006
A	intestine	10.0 ^9^	4 (4.0)	0.40	Lake Choczewskie	2008-15	This study
A	intestine	25.0 ^9^	1 (1.0)	0.25	Lake Kłodno	2015	This study
A	intestine	20.0 ^9^	1 (1.0)	0.20	Lake Dargin	2015	This study
**Family Cystidicolidae**							
*Cystidicola farionis* Fischer, 1798							
-	-	0.7	7 (7)	0.05 ^3^	Puck Bay	2002	Bystydzieńska et al. 2005
*Spinitectus inermis* (Zeder, 1800)							
F	intestine	3.7 ^3^	3 (3)	0.11 ^3^	Baltic Sea (near Chłapowo), Puck Bay	1930-31	Markowski 1933
A, L	intestine	3.3	1-5 (3.0 ^3^)	0.10 ^3^	River Wieprza (near Darłowo)	1999, 2001	Pilecka-Rapacz and Sobecka 2004
A, L	intestine	6.1	1-10 (3.4 ^3^)	0.21 ^3^	River Rega (Lake Rejowice)	1999-2003	Pilecka-Rapacz and Sobecka 2004
A, L	intestine	10.9	1-6 (2.3 ^3^)	0.25 ^3^	River Radew	2000-01	Pilecka-Rapacz and Sobecka 2004
**Family Daniconematidae**							
*Daniconema anguillae* Moravec & Køie, 1987							
-	gills	1.9 ^3^	1 (1.0)	0.02 ^3^	Lake Dąbrowa Wielka	1990-91	Własow et al. 1991
F	sb wall	-	1	-	Reservoir near village Gaj	1993	Własow et al. 1991
**Family Dioctophymatidae**							
*Eustrongylides excisus* Jägerskiöld, 1909							
L	intestine wall	3	3	0.09 ^3^	Szczecin Lagoon	1971	Seyda 1973
L	stomach, body cavity	2.5 ^3^	1-3 (2.2 ^3^)	0.06 ^3^	Szczecin Lagoon	1982-83	Orecka-Grabda and Wierzbicka 1994
**Family Raphidascarididae**							
*Hysterothylacium aduncum* (Rudolphi, 1802)							
-	intestine	33.3 ^3,9^	1 (1.0)	0.33 ^3^	River Gnilna	-	Grabda 1971
-	-	0.2	1 (1)	0.002 ^3^	Vistula Lagoon	2001-02	Bystydzieńska et al. 2005
-	-	0.7	1 (1)	0.01 ^3^	Puck Bay	2002	Bystydzieńska et al. 2005
A	-	2.2	1-2 (1.5)	0.03 ^3^	Vistula Lagoon	2005	Rolbiecki and Rokicki 2006
*Raphidascaris acus* (Bloch, 1779)							
-	intestine	20.0 ^3,9^	1 (1)	0.20 ^3^	Vistula (near Warszawa)	1924-25	Dąbrowska 1970
M	intestine	3.7 ^3^	1 (1)	0.04 ^3^	Baltic Sea (near Chłapowo), Puck Bay	1930-31	Markowski 1933
-	intestine	100 ^3,9^	1 (1.0)	1.00 ^3^	Lake Druzno	1951	Kozicka 1959
-	intestine	-	-	-	River Gnilna, Lake Blanki	-	Grabda 1971
-	-	10	1-2 (1.5 ^3^)	0.15 ^3^	Oder (near Stołczyn)	1971	Seyda 1973
-	-	6	3-4 (3.5 ^3^)	0.22 ^3^	Szczecin Lagoon	1971	Seyda 1973
-	-	13	1-6 (2.5 ^3^)	0.32 ^3^	Lake Dąbie	1971	Seyda 1973
A	-	9.1	1 ^3^ (1 ^3^)	0.09	Lake Dgał Wielki	1979-84	Grabda-Kazubska et al. 1987
A	intestine	2.5 ^3^	1-6 (4.0 ^3^)	0.10 ^3^	Szczecin Lagoon	1982-83	Orecka-Grabda and Wierzbicka 1994
-	-	4.2	2 (2)	0.08	Skolwiński Canal	1982-83	Orecka-Grabda and Wierzbicka 1994
L4, A	intestine	24.28	1-26	0.900	Dead Vistula	1982-90	Sulgostowska 1993
L4, A	intestine	19.06	1-68	1.067	Gulf of Gdańsk	1982-90	Sulgostowska 1993
L4, A	intestine	9.09	1-2	0.107	Baltic Sea (near Władysławowo)	1982-90	Sulgostowska 1993
-	intestine	-	-	-	Lake Ińsko	1993	Rząd and Pilecka-Rapacz 2002
L	intestine	0.4 ^3^	1 (1.0)	0.003 ^3^	Rivers Rega (Lake Rejowice), Radew, Wieprza (near Darłowo)	1999-2003	Pilecka-Rapacz and Sobecka 2004
-	intestine	1.14	1 (1.00)	0.01	Lake Łebsko	2000-06	Morozińska-Gogol 2011
-	-	56.1	1-92 (5.6)	-	Vistula Lagoon	2001-02	Bystydzieńska et al. 2005
-	-	4.5	1-12 (5)	-	Puck Bay	2002	Bystydzieńska et al. 2005
A	intestine	2.2	2 (1.0)	0.02	Lake Wdzydze	2004	This study
**Family Quimperiidae**							
*Paraquimperia tenerrima* (von Linstow, 1878)							
L	intestine	0.4	1	0.0033	Rivers Rega (Lake Rejowice), Radew, Wieprza (near Darłowo)	1999-2003	Pilecka-Rapacz and Sobecka 2004
ACANTHOCEPHALA							
**Family Echinorhynchidae**							
*Acanthocephalus anguillae* (Müller, 1780)							
-	intestine	20.0 ^3,9^	1 (1)	0.20 ^3^	Vistula (near Warszawa)	1924-25	Dąbrowska 1970
-	intestine	55.5 ^3,9^	1-7	-	Potok Oliwski	-	Grabda 1971
-	intestine	33.3 ^3,9^	8	2.67 ^3^	River Łupawa	-	Grabda 1971
-	intestine	36.4 ^3^	1-9	-	Vistula (near Tczew)	-	Grabda 1971
-	intestine	40.0 ^3,9^	1	0.40 ^3^	River Nogat (near Malbork)	-	Grabda 1971
-	intestine	-	-	-	Lake Probark	-	Grabda 1971
-	-	35	1-37 (9.1 ^3^)	3.20 ^3^	Oder (near Stołczyn)	1971	Seyda 1973
-	-	29	1-74 (11.1 ^3^)	3.23 ^3^	Lake Dąbie	1971	Seyda 1973
-	intestine	2.5 ^3^	1-2 (1.2 ^3^)	0.03 ^3^	Szczecin Lagoon	1982-83	Orecka-Grabda and Wierzbicka 1994
-	intestine	4.2	17 (17)	0.71	Skolwiński Canal	1982-83	Orecka-Grabda and Wierzbicka 1994
A	intestine	4.29	1-9	0.157	Dead Vistula	1982-90	Sulgostowska 1993
A	intestine	1.61	1-23	0.061	Gulf of Gdańsk	1982-90	Sulgostowska 1993
-	intestine	43.4	-	-	Lake Ińsko	1993	Rząd and Pilecka-Rapacz 2002
-	intestine	7.95	1-21 (6.57)	0.52	Lake Łebsko	2000-06	Morozińska-Gogol 2011
-	-	0.6	1-8 (3.3)	-	Vistula Lagoon	2001-02	Bystydzieńska et al. 2005
-	-	1.5	1 (1)	0.01 ^3^	Puck Bay	2002	Bystydzieńska et al. 2005
*Acanthocephalus lucii* (Müller, 1776)							
-	intestine	100 ^3,9^	2 (2.0)	2.00 ^3^	Lake Druzno	1951	Styczyńska 1958
-	intestine	100 ^3,9^	2 (2.0)	2.00 ^3^	Lake Druzno	1951	Kozicka 1959
-	intestine	100 ^3,9^	7 (7.0 ^3^)	7.00 ^3^	Lake Wdzydze	1958	Grabda et al. 1961
-	intestine	-	-	-	Vistula mouth, River Łupawa, River Dadaj, Lake Dąbrowa Wielka	-	Grabda 1971
-	-	15	2-8 (4.0 ^3^)	0.60 ^3^	Oder (near Stołczyn)	1971	Seyda 1973
-	-	3	1 (1)	0.03 ^3^	Szczecin Lagoon	1971	Seyda 1973
-	-	10	1 (1)	0.10 ^3^	Lake Dąbie	1971	Seyda 1973
-	intestine	5.0-16.7	1-7 (2.4 ^3^)	0.25 ^3^	Szczecin Lagoon	1982-83	Orecka-Grabda and Wierzbicka 1994
-	intestine	16.7	1-11 (3.8 ^3^)	0.62	Skolwiński Canal	1982-83	Orecka-Grabda and Wierzbicka 1994
A	-	3.57	1-5	0.092	Dead Vistula	1982-90	Sulgostowska 1993
A	-	0.88	1-7	0.024	Gulf of Gdańsk	1982-90	Sulgostowska 1993
-	-	^6^	-	-	Lake Śniardwy	1989	Własow et al. 1991
-	-	^6^	-	-	Lake Mamry	1990	Własow et al. 1991
-	-	^6^	-	-	Lake Dąbrowa Wielka	1990-91	Własow et al. 1991
-	-	0.2	14 (14)	0.03 ^3^	Vistula Lagoon	2001-02	Bystydzieńska et al. 2005
-	intestine	15.91	1-80 (14.14)	2.41	Lake Łebsko	2000-06	Morozińska-Gogol 2011
-	-	0.7	14 (14)	0.11 ^3^	Puck Bay	2002	Bystydzieńska et al. 2005
A	intestine	28.3	1-8 (2.6)	0.74	Lake Wdzydze	2004	This study
A	-	1.1	1	0.01 ^3^	Vistula Lagoon	2005	Rolbiecki and Rokicki 2006
*Echinorhynchus gadi* Zoega in Müller, 1776							
A	-	0.73	1-4	0.014	Gulf of Gdańsk	1982-90	Sulgostowska 1993
-	-	0.2	1 (1)	0.002 ^3^	Vistula Lagoon	2001-02	Bystydzieńska et al. 2005
-	-	3	1-10 (3.2)	-	Puck Bay	2002	Bystydzieńska et al. 2005
*Echinorhynchus truttae* (Schrank, 1788)							
-	intestine	33.3 ^3,9^	3 (3.0)	1.00 ^3^	River Łupawa	-	Grabda 1971
-	intestine	11.1 ^3,9^	1 (1.0)	0.11 ^3^	Potok Oliwski	-	Grabda 1971
-	intestine	3.41	2-21 (8.67)	0.30	Lake Łebsko	2000-06	Morozińska-Gogol 2011
**Family Neoechinorhynchidae**							
*Neoechinorhynchus rutili* (Müller, 1780)							
-	intestine	5.9 ^3^	2	0.12 ^3^	Gulf of Gdańsk	1967-71	Rokicki 1975
-	intestine	2.27	1-2 (1.5)	0.03	Lake Łebsko	2000-06	Morozińska-Gogol 2011
**Family Polymorphidae**							
*Corynosoma strumosum* (Rudolphi, 1802)							
cystacanth	-	1.1	1 (1)	0.01 ^3^	Vistula Lagoon	2005	Rolbiecki and Rokicki 2006
**Family Pomphorhynchidae**							
*Pomphorhynchus laevis* (Zoega in Müller, 1776)							
A	intestine	14.8 ^3^	1-37 (10.8 ^3^)	1.59 ^3^	Baltic Sea (near Chłapowo), Puck Bay	1930-31	Markowski 1933
-	-	3	1 (1)	0.03 ^3^	Lake Dąbie	1971	Seyda 1973
A	intestine	0.44	1-2	0.005	Gulf of Gdańsk	1982-90	Sulgostowska 1993
A	intestine	1.65	1-3	0.033	Baltic Sea	1982-90	Sulgostowska 1993
-	-	6	1-10 (2.8)	-	Puck Bay	2002	Bystydzieńska et al. 2005
-	intestine	3.41	1-2 (1.3)	0.05	Lake Łebsko	2000-06	Morozińska-Gogol 2011
**Family Tenuisentidae**							
*Paratenuisentis ambiguus Van Cleave, 1923*							
A	intestine	6.82	1-163 (28.17)	1.92	Lake Łebsko	2000-06	Morozińska-Gogol (2008), Morozińska-Gogol (2011), Morozińska-Gogol (2009)
MOLLUSCA: BIVALVIA							
**Family Unionidae**							
glochidium	-	9.1	1 ^3^ (1 ^3^)	0.09	Lake Dgał Wielki	1979-84	Grabda-Kazubska et al. 1987
glochidium	-	1.14	21 (21)	0.24	Lake Łebsko	2000-06	Morozińska-Gogol 2011
glochidium	gills	33.3 ^9^	6 (6.0)	2.0	River Słupia	2015-16	This study
ANNELIDA: CLITELLATA							
**Family Piscicolidae**							
*Piscicola geometra* (Linnaeus, 1761)							
-	-	1.1	1	0.01 ^3^	Vistula Lagoon	2005	Rolbiecki and Rokicki 2006
*Piscicola respirans* Troschel, 1850							
-	-	-	-	-	River Dunajec with tributaries	-	Sitowski 1937
ARTHROPODA: BRANCHIURA							
**Family Argulidae**							
*Argulus foliaceus* (Linnaeus, 1758)							
-	gills	1.9 ^3^	1 (1)	0.02 ^3^	Szczecin Lagoon	1982-83	Orecka-Grabda and Wierzbicka 1994
ARTHROPODA: COPEPODA							
**Family Ergasilidae**							
*Ergasilus gibbus* Nordmann, 1832							
-	gills	-	-	-	Vistula Lagoon	-	Zaddach 1844
A	gills	28.5	-	-	Vistula Lagoon	1908	Wegener 1909
-	-	16.6	(1.4)	0.23 ^3^	Lake Dąbie	1955	Kozikowska 1957
-	gills	38.3	1-51 (8.5)	3.21 ^3^	Vistula Lagoon	1959	Grabda 1962
-	-	-	-	-	Puck Bay	1959	Grabda 1962
-	gills	27.6	up to 4 (2.3)	-	Puck Bay	1959	Kozikowska 1965
-	gills	75.0 ^3,9^	up to 75	-	Vistula (near Świbno)	1961-63	Grabda 1972
-	-	-	-	-	Vistula mouth	-	Grabda 1967
-	gills	1.9 ^3^	1-4 (3.0 ^3^)	0.06 ^3^	Szczecin Lagoon	1982-83	Orecka-Grabda and Wierzbicka 1994
*Ergasilus sieboldi* von Nordmann, 1832							
-	gills	38.3	1-12 (3.2)	1.25 ^3^	Vistula Lagoon	1959	Grabda 1962
-	-	-	-	-	Lake Dąbrowa Wielka	-	Grabda 1962
-	-	45.5	(4.4 ^3^)	2.0	Lake Dgał Wielki	1979-84	Grabda-Kazubska et al. 1987
-	gills	52.2 ^3^	1-16 (3.4 ^3^)	1.79 ^3^	Szczecin Lagoon	1982-83	Orecka-Grabda and Wierzbicka 1994
-	gills	33.3	1-4 (2.2 ^3^)	0.75	Skolwiński Canal	1982-83	Orecka-Grabda and Wierzbicka 1994
-	-	^6^	-	-	Lake Śniardwy	1989	Własow et al. 1991
-	gills	10.23	1-23 (7.67)	0.78	Lake Łebsko	2000-06	Morozińska-Gogol 2007, Morozińska-Gogol 2011
-	-	9	1-4 (2.3)	0.20 ^3^	Vistula Lagoon	2005	Rolbiecki and Rokicki 2006
A	gills	100 ^3,9^	(3.0 ^3^)	1.50 ^3^	Lake Żarnowieckie	2007	Rolbiecki 2011
A	gills	10.0 ^9^	3 (3.0)	0.30	Lake Choczewskie	2008-15	This study
A	gills	20.0 ^9^	7 (7.0)	1.40	River Parsęta	2014-16	This study
A	gills	20.0 ^9^	5 (5.0)	1.00	Lake Jasień	2015	This study
A	gills	28.6 ^9^	2-3 (2.5)	0.71	Lake Sarbsko	2016	This study
*Ergasilus* sp.							
-	gills	10	(2)	-	Lake Miedwie	1997-99	Sobecka and Piasecki 2002
^1^ blood, liver, spleen, kidney, skin, necrotic muscles; ^2^ single to numerous in spleen, single to very numerous in blood, liver, kidney; very numerous in skin; ^3^ parameter was calculated on the basis of data from publication; ^4^ parasite number in smear area 20 x 25 mm; ^5^ blood, kidney, liver, urinary bladder, gills; ^6^ rare or sporadic; ^7^ gills, kidney, intestine, urinary bladder, liver, gall bladder, spleen, skin; ^8^ fins, gills, urinary bladder, kidney, liver, intestine; ^9^ calculated from less than ten individuals; ^10^ questionable microhabitat; ^11^ unspecified, the authors provided a value for several reservoirs; ^12^ together Szczecin Lagoon, Vistula Lagoon, Goczałkowicki Reservoir, Lake Charzykowskie, Lake Niegocin, Lake Mamry.							
