# Supplementary material for: Checklist of the parasites of European eel Anguilla anguilla (Linnaeus, 1758) (Anguillidae) in Poland
Source: Biodivers Data J. 2020 Jun 11;8:e52346. doi: 10.3897/BDJ.8.e52346 (PMC7303223; doi:10.3897/BDJ.8.e52346)
Supplement: Supplementary material 1 — GPS coordinates of collection sites [file bdj-08-e52346-s001.docx]

**GPS coordinates in alphabetical order**

| **No.** | **Locality** | **GPS coordinates** |
| --- | --- | --- |
| 1 | Baltic Sea (near Chłapowo) | 54°49'17"N 18°23'21"E |
| 2 | Baltic Sea (near Władysławowo) | 54°48'36"N 18°25'41"E |
| 3 | Dead Vistula | 54°19'31"N 18°50'44"E |
| 4 | Goczałkowicki Reservoir | 49°55'42"N 18°52'05"E |
| 5 | Gulf of Gdańsk | 54°30'33"N 18°56'32"E |
| 6 | Lake Blanki | 54°00'21"N 20°37'41"E |
| 7 | Lake Bukowo | 54°21'11"N 16°17'42"E |
| 8 | Lake Choczewskie | 54°44'16"N 17°55'51"E |
| 9 | Lake Charzykowskie | 53°46'31"N 17°30'36"E |
| 10 | Lake Dąbie | 53°27'02"N 14°40'25"E |
| 11 | Lake Dargin | 54°6'53"N 21°42'38"E |
| 12 | Lake Dąbrowa Wielka (earlier Duża Woda) | 53°26′24″N 20°3′3″ E |
| 13 | Lake Dębno Małe (earlier Gaj) | 53°22'45"N 19°24'53"E |
| 14 | Lake Dgał Wielki | 54°06'31"N 21°47'34"E |
| 15 | Lake Druzno | 54°06'12"N 19°27'43"E |
| 16 | Lake Gardno | 54°39'11"N 17°06'36"E |
| 17 | Lake Gołdapiwo | 54°06'42"N 21°56'59"E |
| 18 | Lake Ińsko | 53°26'39"N 15°32'12"E |
| 19 | Lake Jamno | 54°16'0"N 16°08'46"E |
| 20 | Lake Jasień | 54°20'37"N 18°31'38"E |
| 21 | Lake Kalwa | 53°39'34"N 20°45'58"E |
| 22 | Lake Kłodno | 54°19'16"N 18°06'29"E |
| 23 | Lake Kopań | 54°28'42"N 16°26'48"E |
| 24 | Lake Kuc | 53°49'12"N 21°24'19"E |
| 25 | Lake Łebsko | 54°42'49"N 17°24'31"E |
| 26 | Lake Łętowskie | 54°16'01"N 16°49'32"E |
| 27 | Lake Mamry | 54°10'12"N 21°41'56"E |
| 28 | Lake Miedwie | 53°16'19"N 14°52'55"E |
| 29 | Lake Niegocin | 54°00'00"N 21°46'58"E |
| 30 | Lake Ostrzyckie | 54°15'11"N 18°06'12"E |
| 31 | Lake Probark | 53°49'30"N 21°22'30"E |
| 32 | Lake Przywłoczne | 54°00'04"N 18°03'09"E |
| 33 | Lake Raduńskie Dolne | 54°16'40"N 18°01'33"E |
| 34 | Lake Raduńskie Górne | 54°14'21"N 17°58'37"E |
| 35 | Lake Resko | 53°40'34"N 15°57'57"E |
| 36 | Lake Sarbsko | 54°45'51"N 17°37'51"E |
| 37 | Lake Siecino | 53°36'47"N 16°01'29"E |
| 38 | Lake Skąpe | 53°54'10"N 17°48'47"E |
| 39 | Lake Strażyn | 53°20'07"N 19°26'06"E |
| 40 | Lake Śniardwy | 53°45'23"N 21°44'24"E |
| 41 | Lake Wdzydze | 53°58'34"N 17°54'38"E |
| 42 | Lake Wielewickie | 53°55'22"N 17°50'41"E |
| 43 | Lake Żarnowieckie | 54°46'06"N 18°03'17"E |
| 44 | Oder (near Stołczyn) | 53°29'34"N 14°37'14"E |
| 45 | Oder mouth | 53°31'14"N 14°38'05"E |
| 46 | Pomeranian Bay | 53°57'03"N 14°20'39"E |
| 47 | Potok Oliwski (earlier River Jelitkówka) | 54°25'28"N 18°35'47"E |
| 48 | Puck Bay | 54°39'14"N 18°36'04"E |
| 49 | Reservoir near village Gaj | 52°31'14"N 18°09'19"E |
| 50 | River Dadaj | 53°48'47"N 20°47'49"E |
| 51 | River Darłówka | 54°25'29"N 16°22'58"E |
| 52 | River Drwęca | 53°00'04"N 18°42'16"E |
| 53 | River Dunajec | 50°10'23"N 20°49'13"E |
| 54 | River Gnilna | 54°32'45"N 16°58'44"E |
| 55 | River Łeba | 54°41'11"N 17°27'51"E |
| 56 | River Łupawa | 54°39'59"N 17°03'32"E |
| 57 | River Nogat (near Malbork) | 54°03'03"N 19°02'45"E |
| 58 | River Nogat (near Tczew) | 54°05'49"N 18°48'29"E |
| 59 | River Piąśnica | 54°48'37"N 18°4'3"E |
| 60 | River Parsęta | 54°7'52"N 15°35'6"E |
| 61 | River Radew | 54°03'27"N 15°56'06"E |
| 62 | River Rega (Lake Rejowice) | 53°50'45"N 15°15'43"E |
| 63 | River Rega (near Trzebiatów) | 54°03'52"N 15°15'27"E |
| 64 | River Słupia | 54°22'49"N 17°2'51"E |
| 65 | River Szkarpawa | 54°17'15"N 19°5'42"E |
| 66 | River Węgorapa | 54°12'40"N 21°43'24"E |
| 67 | River Wieprza (near Darłowo) | 54°25'13"N 16°24'23"E |
| 68 | Skolwiński Canal | 53°31'30"N 14°37'01"E |
| 69 | Szczecin Lagoon | 53°46'40"N 14°18'28"E |
| 70 | Vistula (near Świbno) | 54°20'19"N 18°56'29"E |
| 71 | Vistula (near Tczew) | 54°05'50"N 18°48'31"E |
| 72 | Vistula (near Warszawa) | 52°23'44"N 20°47'08"E |
| 73 | Vistula Lagoon | 54°19'27"N 19°22'25"E |
